# Does milk and dairy consumption during pregnancy influence fetal growth and infant birthweight? A systematic literature review

**DOI:** 10.3402/fnr.v56i0.20050

**Published:** 2012-11-23

**Authors:** Anne Lise Brantsæter, Anna S. Olafsdottir, Elisabet Forsum, Sjurdur F. Olsen, Inga Thorsdottir

**Affiliations:** 1 Department of Exposure and Risk Assessment, Division of Environmental Medicine, Norwegian Institute of Public Health, Oslo, Norway; 2 School of Education, University of Iceland, Reykjavik, Iceland; 3 Department of Clinical and Experimental Medicine, Division of Nutrition, Linköping University, Linköping, Sweden; 4 Division of Epidemiology, Centre for Fetal Programming, Statens Serum Institut, Copenhagen, Denmark; 5 Unit for Nutrition Research, Landspitali-University Hospital and University of Iceland, Reykjavik, Iceland

**Keywords:** maternal milk and dairy consumption, fetal growth, systematic review, Nordic Nutrition Recommendations

## Abstract

It is increasingly acknowledged that the maternal diet influences fetal development and health of the child. Milk and milk products contribute essential nutrients and bioactive substances; they are of ample supply and have a long tradition in Nordic countries. To revise and update dietary guidelines for pregnant women valid in Nordic countries, the Pregnancy and Lactation expert group within the NNR5 project identified a need to systematically review recent scientific data on infant growth measures and maternal milk consumption. The objective of this study was to assess the influence of milk and dairy consumption during pregnancy on fetal growth through a systematic review of studies published between January 2000 and December 2011. A literature search was run in June 2011. Two authors independently selected studies for inclusion from the 495 abstracts according to predefined eligibility criteria. A complementary search in January 2012 revealed 64 additional abstracts published during the period June to December 2011, among them one study of interest previously identified. Of the 33 studies extracted, eight were relevant research papers. Five were prospective cohort studies (including a retrospective chart review), one was a case–control study, and two were retrospective cohort studies. For fetal length or infant birth length, three studies reported no association and two reported positive associations with milk or dairy consumption. For birthweight related outcomes, two studies reported no associations, and four studies reported positive associations with milk and/or dairy consumption. There was large heterogeneity in exposure range and effect size between studies. A beneficial fetal growth-increase was most pronounced for increasing maternal milk intake in the lower end of the consumption range. Evidence from prospective cohort studies is limited but suggestive that moderate milk consumption relative to none or very low intake, is positively associated with fetal growth and infant birthweight in healthy, Western populations.

## Introduction

Maternal nutrition during pregnancy is known to have a significant effect on fetal development (1). Mammalian milk is a complex bioactive food and an important vehicle for essential nutrients and endocrine signals to the newborn. No one challenges the health value of breast milk as the optimal nutrition for newborn infants in developed as well as developing countries (2). Cow's milk and dairy products are widely consumed by children as well as by adults in the Western world. Milk is especially recommended in the diet of young children because of its nutritive value. Milk has a high concentration of nutrients including protein, calcium, phosphorus, potassium, iodine, vitamin B12, and riboflavin. It has been shown that the consumption of cow's milk increases blood concentration of insulin growth factor 1 (IGF-1), an important determinant of growth during childhood (3, 4). Because milk and dairy products contribute substantially to the saturated fat intake in Western populations, the general recommendation is to use fat reduced milk and dairy products (5). It has been hypothesized that milk is the promoter of chronic Western diseases (6), but recent systematic reviews found no consistent evidence that milk or dairy food consumption was associated with a higher risk of coronary heart disease or metabolic syndrome (7–9). A recent study from Finland found that a high consumption of milk products during pregnancy was associated with lower risk of cow's milk allergy in the children (10). In general, the Nordic populations have high consumption of milk and dairy products and most individuals maintain the ability to digest lactose throughout life (11).

Low fat milk is recommended as part of a balanced diet during pregnancy in all of the Nordic countries (5). Furthermore, the Nordic populations have higher mean birthweight among newborn infants than most other Western populations (12, 13).

Associations between maternal diet and measures of infant birth size have been reported in numerous studies (14–18). In the wake of the increased awareness of the role of the maternal diet in relation to fetal development and the health of the child, monitoring of dietary intake has become an integral part of pregnancy and birth cohort studies. The focus is shifting from studying the impact of single dietary substances to studying food groups and dietary patterns in relation to pregnancy outcomes. Milk and dairy intake is one of several food groups of special interest during the perinatal period. Recent studies in Nordic populations have reported positive associations between maternal milk consumption and infant birth size (19) and pregnancy weight gain (20). The aim of this systematic literature review was to assess the influence of maternal milk and dairy consumption during pregnancy on fetal growth and infant birthweight.

The present literature review is a part of 5th edition of the Nordic Nutrition Recommendations (NNR5) project with the aim of reviewing and updating the scientific basis of the 4th edition of the NNR issued in 2004 (5). The NNR5 project is mainly focused on a revision of those areas in which new scientific knowledge has emerged since the 4th edition with special relevance for the Nordic setting. A number of systematic literature reviews will form the basis for establishing dietary reference values in the 5th edition of NNR. In addition to updating recommendations with regard to single nutrients, the NNR5 project also intended to update and include more food based dietary guidelines. The experts allocated to the Pregnancy and Lactation group therefore decided to systematically review specific food groups in relation to pregnancy outcome. Among potentially important food groups, we chose milk because of the ample supply and long tradition of milk consumption in all Nordic countries (5).

## Material and methods

### Literature search

The research question was formulated – does milk/dairy consumption in pregnancy have an impact on birthweight and/or fetal growth? – and additional keywords chosen – milk, dairy, birthweight, fetal growth, large for gestational age (LGA), small for gestational age (SGA), and intra uterine growth retardation (IUGR). Criteria for inclusion and exclusion were also defined prior to the search. Only papers relevant to the research question and those dealing with humans, healthy Western women, published in 2000 or later were to be included.

Librarians Jannes Engqvist and Mikaela Bachman of the Swedish National Food Agency conducted the literature research, with support from Sveinn Ólafsson from the National and University Library of Iceland in June 2011. The databases used were PubMed/Medline, provided by the American National Library of Medicine and SweMed+.

Search terms included (milk[Title/Abstract] OR milk products[Title/Abstract] OR dairy[Title/Abstract] OR dairy products[Title/Abstract] OR “Cultured Milk Products”[Mesh] OR “Dairy Products”[Mesh]) AND (eating[Title/Abstract] OR intake[Title/Abstract] OR consumption[Title/Abstract] OR “Eating”[Mesh] OR “Drinking”[Mesh] OR Drinking[Title/abstract]) AND (“pregnancy”[Title/Abstract] OR “Pregnancy”[Mesh] OR “Infant, Low Birth Weight”[Mesh] OR “Fetal Growth Retardation”[Mesh] OR “Birth Weight”[Mesh] OR “Fetal Development”[Mesh] OR “Birth Weight”[Title/abstract] OR “Fetal Growth”[Title/Abstract] OR LGA[Title/Abstract] OR large for gestational age[Title/Abstract] OR SGA[Title/Abstract] OR small for gestational age[Title/Abstract] OR IUGR[Title/Abstract] OR intra uterine growth retardation[Title/Abstract]) AND (“2000/01/01”[PDAT]: “2011/05/31”[PDAT]) AND (English[lang] OR Danish[lang] OR Finnish[lang] OR Icelandic[lang] OR Norwegian[lang] OR Swedish[lang]) NOT (“Asia”[Mesh] OR “Africa”[Mesh] OR “South America”[Mesh] OR “Central America”[Mesh] OR “Mexico”[Mesh] OR “Latin America”[Mesh] OR “Caribbean Region”[Mesh]) NOT (“animals”[MeSH Terms:noexp] NOT “humans”[MeSH:noexp]).

### Selection of articles

Two experts reviewed the list of abstracts independently and a paper was ordered in full text if one of the experts chose to include it. Non-human subjects were excluded as well as papers investigating women that were not healthy or Western. However, all subpopulations living in the United States were included. Abstracts not seen as relevant for ordering the article in full text were excluded and the reasons for exclusion were written down. A list of articles selected for full text screening was sent to the librarian who ordered the articles in full text. One additional abstract published and identified in June 2011 was also ordered in full text.

The full text papers were split into two groups and two independent experts reviewed each paper. The experts jointly decided which papers to include for quality assessment and grading. The excluded articles were listed with reasons for exclusion (Appendix).

A complementary search was conducted at the end of January 2012 covering the period between the end of the first search up to the end of December 2011. The search was performed using the same search strings as in the first search.

### Quality assessment and grading of evidence

To assess and rate the quality of the included studies, we applied a three-category (A–B–C) grading system based on the NNR5 AMSTAR quality assessment tool (QAT). Assessment tools applicable to each type of study design were provided. These include questions for evaluating all aspects of a study (e.g. study design, population characteristics, assessment of dietary exposures and relevant outcomes, confounding factors, power, and analyses).

Two experts assessed the quality of the same studies independently and disagreement between experts was discussed by all Pregnancy and Lactation experts. The quality was evaluated, graded, and summarized into one of four categories: convincing (high), probable (moderate), limited – suggestive (low) and limited – no conclusion (insufficient). This system was developed in the 2007 World Cancer Report to also take into account evidence from observational studies (21).

## Results

The original search retrieved 495 abstracts from which 32 full text papers were selected and ordered. One additional full text paper was published after the systematic search was performed and subsequently added (23). The complementary search identified 64 abstracts (including the paper from Heppe et al.), but none of them were considered relevant for full text evaluation. No intervention studies were identified.

Of the 33 full text papers, 25 were excluded (Appendix). Nine retrieved studies were not research papers and the rest did not report maternal milk/dairy as exposure or fetal growth measures among their outcomes. Eight of the 33 full text papers were research papers relevant to the research question, of which five were prospective cohort studies (including a retrospective chart review), one was a case–control study, and two were retrospective cohort studies (Table 1). All studies reported the variables to be considered as confounders, that is anthropometric, sociodemographic, and/or lifestyle characteristics of the subjects, but did not report sufficient detail for comparison of, for example, educational level between studies. The eight studies comprised more than 100,000 pregnant women, of which 65% were from Denmark, Sweden, or The Netherlands, 33% were from North America, and 1% was from Australia and New Zealand (Table 1).

**Table 1:** Description of the eight included studies comprising five prospective cohorts, one case–control and two retrospective studies

Reference	Cohort, country and type of study	No. of participants, year of study	Age	Exposure	Diet method	Outcome	Follow-up	Confounder adjustments	Quality
Chang et al. (22)	Retrospective chart review within a prospective cohort of African American adolescents, USA.	N=350 Years: 1990–2000.	≤17 y Mean (SD) 15.9 (1.1)	Dairy intake, 5 categories, reduced into three levels of intake (high, medium, low) for further analysis.	24-h dietary recall and FFQ combined.	Fetal femur length, birthweight, fetal biparietal diameter, fetal head circumference and fetal abdominal circumference.	None	Gestational age, biparietal diameter, maternal age and height, prepregnancy BMI. Association with total energy and other nutrients considered.	B
Heppe et al. (23)	Prospective cohort study, Netherlands.	N=3,405 Years: 2001–2005.	Mean (SD) 31.4 (4.4)	Primary: Milk consumption (glasses/day) Secondary: macronutrients from milk/dairy.	FFQ	Fetal head circumference, femur length and fetal weight estimated by ultrasound, and birthweight, length and head circumference.	Measurements obtained at second and third trimester and at birth	A range of relevant maternal characteristics and other dietary intakes, including total energy.	B
Mannion et al. (24)	Prospective cohort study, Canada.	N=279 Years: 1997–1999.	19–45 y, mean age 31 y.	Milk consumption (≤250 ml vs. >250 ml/day.	Repeated 24 h telephone recalls	Infant birthweight, crown–heel length and head circumference.	None	Maternal characteristics including gestational weight gain. Other foods/nutrients also considered.	B
Moore et al. (26)	Prospective cohort study, Australia.	N=557 Years: 1998–2000.	18–41 y, mean: 29 y.	Percentage of protein from dairy.	Face-to-face interviews using semiquantitative FFQ early and late in pregnancy.	Infant birthweight (and ponderal index).	None	Maternal age, anthropometric data, gestational weight gain, parity, smoking, alcohol and drug use.	B
Olsen et al. (19)	Prospective cohort study, Denmark.	N=50,117 mother–infant pairs Years: 1996–2002.	30.4 y in women reporting no milk consumption and 28.2 y in the high consumption group.	Milk consumption by 8 categories (glasses/day) and protein from total dairy consumption (g/day).	FFQ in week 25 of gestation.	Birth length and weight. Risk of SGA and LGA (+abdominal circumference, placental weight, head circumference).	None	Gestational age, infant's sex, maternal age, height, BMI, parity, gestational weight gain, smoking, energy intake, paternal height and family socioeconomic status.	B
Mitchell et al. (25)	Case–control study, New Zealand.	N=1,714 divided into 844 cases (SGA) and 870 controls (AGA). Years: 1995–1997.	Mean age cases: 29.1 y, controls: 30.3 y.	Focus on different foods and supplements including dairy. Dairy products, servings per day in 5 groups.	FFQ early and late in pregnancy, collected retrospectively.	Differences in dairy intake between mothers of SGA and AGA babies.	None	Socioeconomic status, ethnicity, maternal height, weight, hypertension and smoking.	B
Ludvigsson & Ludvigsson, (27)	Retrospective cohort, Sweden.	N≈14,000 mother–infant pairs Years: 1997–1999.	Age not reported but included among confounders.	Milk consumption, (dl/day) divided into 4 groups.	Questionnaire including questions about milk intake completed just after birth.	Low birthweight, IUGR and preterm birth.	None	Sex of infant and a range of maternal characteristics, alcohol intake, major food intakes and supplements.	C
Xue et al. (28)	Retrospective cohort, USA.	N=34,063 mothers of nurses Year: 2001–2002 (data collection).	Age at time of delivery 26.3 y in mothers of babies with birthweight <2,500 g and 27.2 y in mothers of babies with birthweight ≥4,000 g.	Milk consumption in glasses/day divided by 4 categories.	A questionnaire was mailed to the mothers in 2001–2002 asking about consumption of common energy- and nutrient dense foods during their index pregnancy decades earlier.	Birthweight and IUGR.	None	Parental anthropometric characteristics, pregnancy conditions, parental behavior, maternal diet during index pregnancy, parental demographic and socioeconomic characteristics, maternal reproductive factors.	C

No studies were rated quality A, six studies were rated quality B and two studies were rated quality C. The main biases identified pertained to missing information regarding the dietary exposure, discussion of measurement error, lack of power calculations, and sensitivity analyses. The dietary assessment methods, time of administration, time period covered, and exposure range differed between the studies (Table 2). All papers reported the exposure by volume or servings of milk/dairy, but a direct comparison of the average daily consumption of milk and milk products was not possible due to lacking information in some of the papers. However, the ranges of reported intake categories indicated that the exposure varied considerably between studies (Table 2).

**Table 2:** Description of the dietary assessment methods, time period covered, exposure quantity and range in the studies

Reference	Study design	Diet method and time of assessment	Time period covered	FFQ validation	Exposure and average intake	Range of exposure
Chang et al. (22)	Retrospective chart review within a prospective cohor.	24-h dietary recall and FFQ administered by a registered dietician (RD) at first prenatal visit.	Habitual intake according to a combination of 24 h recall and FFQ.	No validation but RD discussed the FFQ with the participants.	Dairy intake, servings/day divided into 5 categories, further reduced into high (≥3), medium (2–<3) and low (<2) intake. No average consumption was reported, but 51% of women were in the low intake group (<2 serving/day) One serving was ≈300 mg Ca, which ≈260 ml milk ≈1 glass.	0 –<1 serving/day 1 –<2 servings/day 2 –<3 servings/day 3 –<4 servings/day ≥ 4 servings/day
Heppe et al. (23)	Prospective cohort.	Modified version of a semi-quantitative FFQ, at enrolment (median 13.5 weeks of gestation). Additional questions on milk and milk products.	Previous 3 months, covering intake within the first trimester.	The FFQ had been validated in elderly, non-pregnant population.	Primary: Milk consumption (glasses/day) in 4 categories. Glass ≈150 ml. Median milk consumption was 2.6 glasses/day (interquartile range 2.1 glass/day) Secondary: macronutrients from milk/dairy.	0–1 glass/day >1 – 2 glasses/day >2 – 3 glasses/day >3 glasses/day
Mannion et al. (24)	Prospective cohort.	Repeated 24 h recalls (3–4 times) by trained interviewers using cups, plates, bowls and rulers for estimating quantity.	Habitual intake.	Method previously validated.	Milk consumption by two categories. The study recruited women who restricted their milk intake. Average milk consumption was not reported.	≤250 ml/day (≤1 glass) >250 ml/day (>1 glass)
Moore et al. (26)	Prospective cohort.	FFQ carried out in an interview early (<16 w) and late in pregnancy (30–34 weeks of gestation).	Early and late pregnancy.	The FFQ was validated in a sub-sample of the cohort.	Protein intake was divided into cereal, meat and dairy sources. Average consumption of protein from dairy was not reported.	Energy% contributed by protein from dairy
Olsen et al. (19)	Prospective cohort.	A 360 item FFQ completed ≈week 25 of gestation referring to the previous 4 weeks.	Previous 4 weeks, ≈ week 21–25 in pregnancy.	The FFQ was validated in a subsample of the cohort, but not specifically for milk or milk components.	Primary: Milk consumption (glass ≈200 ml/day) and yoghurt (portion ≈150 ml/day) aggregated into glasses/day in 8 categories (cheese and ice-cream were excluded). Average milk consumption was 3.1 ±2.0 glasses/day. Secondary: Protein from total dairy consumption (g/day).	0 >0–1 glass/day >1–2 glasses/day >2–3 glasses/day >3–4 glasses/day >4–5 glasses/day >5–6 glasses/day >6 glasses/day
Mitchell et al. (25)	Case–control study.	FFQ early and late in pregnancy, collected retrospectively shortly after birth. Total daily/weekly intake calculated as servings and divided into seven food groups including dairy (milk, cheese and yoghurt).	Time of conception and last month of pregnancy.	91 women were recruited early in pregnancy for comparison of FFQ filled out retrospectively for that time period.	Dairy products in servings/day divided into in 5 categories. The median consumption was 2.2 serving/day in mothers of small for gestational age babies, and 2.5 servings/day in mothers of appropriate size for gestational age babies.	0–1.25 >1.25–2.0 >2.0–3.0 >3.0–4.0 >4.0
Ludvigsson & Ludvigsson (27)	Retrospective cohort.	Mothers received a questionnaire about milk intake shortly after birth.	During pregnancy.	No information.	Milk consumption (dl/day) divided into 4 predefined categories. Milk comprised of milk, yoghurt and sour milk. Average milk consumption or distribution by consumption categories were not reported.	No consumption ≤2 dl/day 3–10 dl/day >10 dl/day
Xue et al. (28)	Retrospective cohort.	Questionnaire mailed to mothers 2001–2002.	Recall of diet during pregnancy decades earlier.	No information.	Milk consumption in glasses/day (continuous) and 4 categories. Average milk consumption was not reported, but 60% of women were in the lower intake groups (≤1 glasses/day).	≤4 glasses/week 5 glasses/w – 1 glass/day 2–3 glasses/day ≥4 glasses/day.

## Effects of exposure on outcome measures

### Fetal femur length and infant birth length

Four studies reported results of maternal dairy or milk consumption in relation to fetal femur length and/or birth length. In two studies, one retrospective chart review and one prospective cohort, the outcome was ultrasound measurements of fetal femur length obtained at several time points during pregnancy (22, 23) (Table 3). Chang et al. found that dairy intake had a significant positive effect on fetal femur growth after adjustment for gestational age, biparietal parameter, maternal age, height, and prepregnancy body mass index (BMI) (22). Contrary to this, Heppe et al. found no association between maternal milk consumption and femur length or estimated fetal length (23). This latter study also found no effect of milk consumption on infant length at birth.

Infant crown–heel length at birth was examined among infant growth measure outcomes in two other studies. Mannion et al. found no influence of low milk consumption on infant length (24), while Olsen et al. found that milk intake was positively associated with increased length at birth in the large Danish National Birth Cohort (19) (Table 3).

**Table 3:** Evidence table for outcome

Reference	Study design	Exposure	Outcome measures	No. of subjects analyzed	Results
Fetal femur length, fetal length, and infant birth length					
Chang et al. (22)	Retrospective chart review within a prospective cohort.	Dairy intake at first prenatal visit estimated on the basis of number of servings of dairy products and rated as calcium intake.	Fetal femur length by ultrasound in 2nd and 3rd trimester.	350	Fetal femur length lower in lowest dairy-intake group (<2 servings/day) than highest group (>3 servings/day) p<0.001, and a dose response suggested.
Heppe et al. (23)	Prospective cohort study.	Maternal dietary intake, including milk and dairy consumption.	1. Femur length by ultrasound in 2nd and 3rd trimester. 2. Infant length at birth.	3,405	Milk consumption in 4 categories based on the distribution of milk consumption (0–1, >1–2, >2–3, >3 glasses/day). No consistent associations with fetal length or infant length at birth.
Mannion et al. (24)	Prospective cohort study.	Milk consumption (≤250 ml vs. >250 ml/day. With one cup (250 ml) giving approximately 300 mg Ca and 90 IU vitamin D.	Infant length at birth.	279, of which 72 restricted daily milk intake to 250 ml or less and 207 with milk intake>250 ml/day.	No influence of milk consumption on infant length at birth.
Olsen et al. (19)	Prospective cohort study.	FFQ answered at 25 wk of gestation, covering intake the previous 4 weeks.	Infant length at birth.	50,117.	Milk intake was positively associated with increased length at birth (also abdominal and head circumferences) across the intake range, with total increment of 0.31 cm (95% CI: 0.15, 0.46 cm).
Infant birthweight, SGA, LGA, IUGR					
Heppe et al. (23)	Prospective cohort study.	Maternal dietary intake, including milk and dairy consumption.	1. Estimated fetal weight based on ultrasound measures in 2nd and 3rd trimester. 2. Birthweight.	3,405	Milk consumption in 4 categories based on the distribution of milk consumption (glasses/day). No association between milk consumption and estimated fetal weight, but a significant positive association with infant birthweight (p<0.01). Birthweight difference between the highest and lowest categories of milk consumption was 88 g (95% CI: 39, 135). The results suggest that the growth-promoting effect was driven by milk protein.
Mannion et al. (24)	Prospective cohort study.	Milk consumption (≤250 ml vs. >250 ml/day. With one cup (250 ml) giving approximately 300 mg Ca and 90 IU vitamin D.	Birthweight.	279, of which 72 with daily milk intake restricted to 250 ml or less and 207 with milk intake>250 ml/day.	For each cup of milk consumed per day, birthweight increased by 41 g (95% CI: 13, 75 g). Women who restricted milk intake had lower vitamin D intake as milk is enriched with vitamin D in Canada, vitamin D was positively associated with birthweight.
Moore et al. (26)	Prospective cohort study.	Protein contributed by dairy assessed by FFQ early and late in pregnancy.	Birthweight (also placental weight and ponderal index).	557, significant result for the exposure only obtained in 429 participants with reliable dietary data.	Each isoenergetic 1% increase in dairy protein consumption in early pregnancy was associated with a 25 g increase in birthweight (p=0.02) and a 0.12 kg/m^3^ increase in ponderal index (p=0.05). Dairy protein was associated more strongly with birth size than other protein sources.
Olsen et al. (19)	Prospective cohort study.	FFQ answered at 25 wk of gestation, covering intake the previous 4 weeks.	1. Infant birthweight, 2. Risks of SGA and LGA.	50,117	Mean birthweight was 108 g (95% CI: 74, 143 g) higher among the group that consumed >6 glasses of milk/day compared with those who consumed no milk. The odds of SGA declined with increasing consumption of milk compared with women who never consumed milk. Women consuming >6 glasses/day had a 49% (95% CI: 35, 61%) lower adjusted odds of SGA. Simultaneously they had 59% (95% CI: 16, 116%) higher odds of LGA. Birthweight also had an association with protein from dairy products, but was constant when considering non-dairy protein.
Mitchell et al. (25)	Case–control study.	Retrospective FFQs completed at birth asking for intake in early and late pregnancy of dairy products (milk, cheese and yoghurt) and six other food groups.	Differences in dairy intake between mothers of SGA and AGA babies.	1,714 of which 844 were mothers of SGA babies and 870 mothers of AGA babies.	In unadjusted analysis SGA mothers reported lower intake of dairy products (only a trend, p=0.05–0.056) but no differences were found for dairy intake at early or late pregnancy in adjusted analyses.
Ludvigsson & Ludvigsson, (27)	Retrospective cohort study.	Milk consumption, (dl/day) divided into 4 groups.	Risk of low birthweight (LBW) and IUGR.	∼14,000	Low milk intake in pregnancy was associated with increased risk of IUGR (p=0.019) when adjusted for confounders, but not with LBW. In unadjusted analysis, the mean birthweight increased with milk consumption in a dose-response manner (p<0.001), with infants of mothers consuming >10 dl/day being 134 g (95%CI: 27, 241 g, p=0.006) heavier than those of mothers consuming no milk and 75 g (95% CI: 18, 123 g, p=0.003) heavier than those of mothers consuming ≤2 dl/day.
Xue et al. (28)	Retrospective cohort study.	Maternal intake of milk assessed by a questionnaire that asked about intake of major foods.	Birthweight of nurse daughter recalled by mother, and IUGR.	34,063	Daily consumption of each additional glass of milk was associated with an increase of ∼6 g in birthweight (p=0.01). Consumption of 2–3 and 4+ glasses of milk/day was associated with a 16 g (p=0.007) and a 19 g (p=0.13) increase respectively, when compared with ≤4 glasses/week (p=0.01). The dose-response was more consistent among term than preterm deliveries, and the association was stronger for non-smokers than smokers. IUGR was not related to maternal milk consumption.

### Fetal or infant birthweight, SGA or LGA infant, and IUGR

Seven of the eight studies reported results of maternal dairy or milk consumption in relation to infant birthweight or other outcomes based on birthweight. Of these, one was a case–control design (25), four were prospective cohorts (19, 23, 24, 26), and two were retrospective cohorts (27, 28) (Table 3).

Mitchell et al. (25) found lower dairy consumption in women who delivered SGA babies than in controls, but the differences disappeared when adjusting for maternal height, weight, smoking, hypertension, and socioeconomic status. No consistent association was found for the fetal weight outcome estimated from ultrasound measures (23). However, all four prospective studies reported a positive association between maternal milk or dairy intake (or protein from milk) and birthweight (Table 3). Heppe et al. reported a birthweight difference between the highest and lowest categories of milk consumption of 88 g, Mannion et al. (24) reported a 41 g increase in birthweight for each daily cup of milk, Moore et al. (26) reported a 25 g increase in birthweight for each isocaloric increase in dairy protein consumption, and Olsen et al. (19) reported a birthweight difference between the highest and lowest categories of milk consumption of 108 g. This last study also found that the positive association between milk consumption and birthweight could be explained by protein from dairy products. Olsen et al. also examined the risk of giving birth to SGA or LGA babies. Milk consumption was inversely associated with the risk of SGA and positively associated with the risk of LGA. Compared with women who reported never consuming milk, women consuming more than six glasses per day had 49% (95% CI: 35%, 61%) lower adjusted odds of having an SGA infant. The corresponding risk increase was 59% (95% CI: 16%, 116%) for having an LGA infant.

Of the retrospective cohorts, one found a positive association between milk consumption and birthweight in crude analyses only, but found a significantly increased risk of IUGR associated with low milk intake (27). The other retrospective cohort study found a significant positive dose-response for maternal milk consumption and infant birthweight (28) (Table 3).

### Reporting and summarizing the evidence

A summary of the evidence is presented in Table 4. The quality of the evidence was graded *limited to no conclusion* for fetal and infant length and *limited – suggestive* for infant birthweight.

**Table 4:** Summary table of associations between maternal milk intake and infant growth measures

Outcome variable	Exposure	Study design: participants (no of studies)	Association/effect	Number of studies rated A, B or C	Strength of evidence
Fetal femur length	Maternal milk/dairy intake	Cohorts prospective: 3,755 (2)	Positive (1) or NS (1)	2 rated as B	Limited – no conclusion
Infant length	Maternal milk/dairy intake	Cohorts prospective: 53,801 (3)	Positive (1) or NS (2)	3 rated as B	Limited – suggestive
Birthweight	Maternal milk/dairy intake and/or milk/dairy protein	Cohorts prospective: 54,358 (4)	Positive (4)	4 rated as B	Limited – suggestive
Birthweight	Maternal intake of protein from milk	Cohorts retrospective cohorts ∼48,000 (2)	Positive (1) NS (1)	2 rated as C	Limited – no conclusion
SGA	Maternal milk/dairy intake	Case–control: 1,714 (1)	NS (1)	1 rated as B	Limited – no conclusion
SGA	Maternal milk/dairy intake	Cohort prospective 50,113 (1)	Positive (1)	1 rated as B	Limited – suggestive
LGA	Maternal milk/dairy intake	Cohort prospective 50,113 (1)	Positive (1)	1 rated as B	Limited – suggestive
IUGR	Protein from milk/dairy	Cohort retrospective ∼14,000 (1)	Positive (1)	1 rated as C	Limited – no conclusion

## Discussion

In this systematic review of studies published during 2000–2011, two prospective studies were suggestive of a positive association between maternal milk consumption and fetal femur length or infant birth length, and four prospective studies were suggestive of a positive association with infant birthweight. However, the number of studies was low and methodological issues in relation to the exposure assessment limited their quality. All the included studies reported either a positive or a no-association between the exposure and outcomes studied. It cannot be ruled out that publication bias might be responsible for the distribution of positive and neutral studies and the subsequent lack of negative findings. Bias and confounding imprecision were important criteria in the quality assessment of individual studies, but imprecision and bias related to the dietary assessment as well as other biases may still have influenced the results. There was large heterogeneity between the studies with regard to the dietary method, time covered by the dietary method, and range of exposure (Table 2). Large variability in the exposure data will most likely lead to attenuation, or an underestimated association with the outcome.

Two prospective studies reported a significant influence of milk intake on fetal femur length or infant birth length, while two prospective studies reported no associations (Table 3). One of the prospective studies reporting a positive association was conducted as a retrospective chart review in an African–American adolescent population (age ≤ 17 years) and it could be questioned whether this study is relevant for comparison with Nordic women. However, as the large Danish cohort reported a positive association, we concluded that the evidence for a potential association between milk consumption during pregnancy and infant length growth is limited but suggestive.

No randomized controlled trials in human, Western, and healthy women were identified in the 10-year window intended for this systematic review. To the best of our knowledge, only one randomized controlled study has examined the relationship between maternal intake of cow's milk and measures of infant birth size (29). That was a study set up in Wales in 1972 to investigate the growth of children, from early in pregnancy to age five. Pregnant women were randomly assigned to either receive or not receive tokens entitling them to a pint of milk at half price each day. No statistically significant differences in birthweights were found between the groups. The authors reported that the trial could be criticized because the issuing of tokens were delayed and each woman only received them for about half of the pregnancy, and consequently it was doubtful whether the intervention actually resulted in any increased milk intake in the pregnant women. However, according to personal communication, Olsen et al. reported that reanalysis of the data from Wales (intention to treat) showed a mean increase in birth weight of 53 g among infants to mothers in the intervention group (95% CI: −6, 111 g).

Among the included studies, the large prospective study from Denmark (*n*=50,117 mother–infant pairs) is the most valuable evidence of a positive influence of maternal milk consumption on infant growth measures. Maternal milk consumption in mid-pregnancy was inversely associated with the risk of SGA at birth, and positively associated with both LGA at birth and mean birth length and weight (19). The strengths of that study were the large sample size, the prospective design, application of a Food Frequency Questionnaire (FFQ) developed and validated for use in the target population, and information about a wide range of potential confounding factors. The confounding variables in the adjusted analyses included maternal age, height, prepregnant BMI, parity, gestational weight gain, smoking status, total energy intake, father's height, and socioeconomic status. This study did not evaluate the role of dietary calcium due to high collinearity of milk and total dietary calcium intakes. However, protein intake from cow's milk was associated with birthweight, whereas non-dairy protein was not, thus making a general protein effect less likely.

Another large cohort study that reported a significant positive association between maternal milk intake and infant birthweight was the Nurses’ Mother's Cohort comprising 34,063 nurses and their mothers (28). The mothers of the nurses were asked to recall the birthweight of their nurse daughter as well as a variety of questions related to early life exposures of their nurse daughter, including consumption of common energy and nutrient dense foods during pregnancy. Due to the retrospective design and long recall period of exposures, this study was rated as a lower quality study than the prospective Danish cohort.

Three smaller prospective studies also reported positive associations between maternal milk or dairy consumption and birthweight-related measures (23, 24, 26). Together with the Danish study, these studies were considered sufficient proof to suggest a positive influence of maternal milk consumption on fetal growth. Comparison of the reported effect size (weight increase) is difficult across the studies, but the reported growth increase was most prominent for increases in the lower end of the consumption range. In the Danish study, the mean birthweight was 48 g higher in women who consumed up to one glass of milk per day than in non-consumers. The corresponding increase was 57 g for consuming one to two glasses/day, and 66 g for consumption of two to three glasses per day. Increased risk of LGA was observed for intakes beyond two glasses of milk per daily (19).

In the analyses where macronutrients intake from dairy products was used as the exposure, protein, but not fat or carbohydrate from milk, was associated with higher birthweight in three of the included studies (19, 23, 26). The Canadian study reported that in addition to milk intake, intake of vitamin D was also an independent predictor of increased birthweight. In Canada, all milk is fortified with vitamin D and women who restricted their milk intake had lower intakes of protein and vitamin D (24). Many factors in milk could promote fetal growth, but collinearity among dietary constituents such as intake of milk, milk protein, and dietary calcium makes it difficult to evaluate the role of single nutrients such as vitamin D and calcium. The fact that milk protein and not non-dairy protein was associated with infant birth measures in the Danish cohort (19) suggests that the association could not be explained by a general protein effect. The authors suggested that another potential explanation could be the peptide hormone IGF-I or other peptide hormones, since milk consumption has been shown to be associated with higher blood concentration of IGF-I both in children and adults (19). They stated that their results support the hypothesis that water-soluble substances in milk increase fetal growth. However, as milk intake was associated not only with decreased risk of SGA but also with increased risk of LGA, the authors did not conclude that the growth-stimulating effect of cow's milk was clearly beneficial but that more research is needed to identify causative factors and overall health implications.

All studies in the review included potential confounding factors in their analyses, but confounding by factors not included may still have influenced the results. Some of these may be considered as potential confounding factors or as mediating factors, e.g. maternal pre-pregnant BMI and gestational weight gain, which are both related to maternal dietary behavior and infant growth measures. Birthweight is highly correlated with gestational weight gain (30–32). A study from Iceland identified milk intake among predictors of optimal and also excessive weight gain during pregnancy, but since associations with birth outcome were only considered for weight gain and not milk intake per se, it was not included in the current systematic review (20). The association between high milk consumption and excessive weight gain in this study and increased risk of LGA in the Danish cohort (19) indicate that milk consumption beyond the general moderate recommendation of two to three daily servings may not be favorable. However, more studies are needed.

The studies included in the review differed with regard to assessment of maternal milk and dairy intake. Two used 24-hour recalls (22, 24), while the remaining applied various FFQs. Studies rated as C were particularly inadequate in their description of dietary exposure (27, 28). Furthermore, it should be noted that the positive influence of milk protein on birthweight in the Australian study (26) was only observed in a subset of participants judged to have reliable data based on pre-described criteria including the plausibility of dietary data relative to estimated energy expenditure.

Among the studies not included, several reported associations between milk or dairy consumption (or milk related components) and preeclampsia and preterm delivery (Appendix), outcomes that usually result in babies having lower than expected birthweight. However, these studies were not included because they did not report infant growth measures as defined outcomes and we had restricted the systematic review to include healthy women only.

The weight increasing influence of milk or dairy consumption varied in the prospective studies (Table 3) and it could be questioned whether the lower birthweight observed in infants of women with low milk intake in the Canadian study (24) is of clinical relevance. That study was also criticized for overrepresentation of women who restricted their milk intake (33).

In conclusion, the evidence from studies published between 2000 and 2011 is limited but suggestive of a positive association between milk consumption and fetal growth and infant birthweight in healthy, Western populations. It should be noted that excessive milk intake was associated with an increased risk of LGA in a large prospective study, and that the potentially beneficial influence of milk and dairy on fetal growth was observed for moderate intake relative to none or a very low intake. In spite of the limited evidence, nearly all papers highlighted the importance of including some milk and dairy in the maternal diet as a source of protein and other valuable nutrients.

## Appendix

**Table:** Full text papers identified in the screening of abstracts but not included in the final review – reasons for exclusion

Papers excluded	Reason for exclusion
Bronner YL, Hawkins AS, Holt ML et al. Models for nutrition education to increase consumption of calcium and dairy products among African Americans. J Nutr 2006;136:1103–6.	Not a research paper
Derbyshire E. The value of consuming a calcium-rich diet: a focus on pregnancy. Br J Nurs 2008;17:856–8.	Not a research paper
Di Cintio E, Parazzini F, Chatenoud L et al. Dietary factors and risk of spontaneous abortion. Eur J Obstet Gynecol Reprod Biol 2001;95:132–6.	Fetal growth measures not reported among outcomes
Duvekot EJ, de Groot CJ, Bloemenkamp KW, Oei SG. Pregnant women with a low milk intake have an increased risk of developing preeclampsia. Eur J Obstet Gynecol Reprod Biol 2002;105:11–4.	Fetal growth measures not reported among outcomes
Frederick IO, Williams MA, Dashow E, Kestin M, Zhang C, Leisenring WM. Dietary fiber, potassium, magnesium and calcium in relation to the risk of preeclampsia. J Reprod Med 2005;50:332–44.	Fetal growth measures not reported among outcomes
Giroux I, Inglis SD, Lander S, Gerrie S, Mottola MF. Dietary intake, weight gain, and birth outcomes of physically active pregnant women: a pilot study. Appl Physiol Nutr Metab 2006;31:483–9.	The main exposure examined was physical activity
Goldstein DA, Kowalczyk DF, Vicini JL, Buonomo FC, Farmer DR. Twinning and higher intake of dairy products. J Reprod Med 2007;52:140–1.	Fetal growth measures not reported among outcomes
Groziak SM, Miller GD. Natural bioactive substances in milk and colostrum: effects on the arterial blood pressure system. Br J Nutr 2000;84 Suppl 1:S119–S125.	Not relevant to the research question
Javaid MK, Crozier SR, Harvey NC et al. Maternal and seasonal predictors of change in calcaneal quantitative ultrasound during pregnancy. J Clin Endocrinol Metab 2005;90:5182–7.	Not relevant to the research question
Knudsen VK, Orozova-Bekkevold IM, Mikkelsen TB, Wolff S, Olsen SF. Major dietary patterns in pregnancy and fetal growth. Eur J Clin Nutr 2008;62:463–70.	Not relevant to the research question - exposure
McCarron DA, Heaney RP. Estimated healthcare savings associated with adequate dairy food intake. Am J Hypertens 2004;17:88–97.	Not a research paper
McCowan LM, Roberts CT, Dekker GA et al. Risk factors for small-for-gestational-age infants by customised birthweight centiles: data from an international prospective cohort study. BJOG 2010;117:1599–607.	Not a research paper
Mehta T. Milk intake in pregnancy. CMAJ 2007;176:1460.	Letter to the editor, comment to a study included in the review: Mannion et al. 2007
Melnik BC. Milk–the promoter of chronic Western diseases. Med Hypotheses 2009;72:631–9.	Not a research paper
Melnik BC. Permanent impairment of insulin resistance from pregnancy to adulthood: the primary basic risk factor of chronic Western diseases. Med Hypotheses 2009;73:670–81.	Not a research paper
Melnik BC. Milk signalling in the pathogenesis of type 2 diabetes. Med Hypotheses 2011;76:553–9.	Not a research paper
Millward DJ, Garnett T. Plenary Lecture 3: Food and the planet: nutritional dilemmas of greenhouse gas emission reductions through reduced intakes of meat and dairy foods. Proc Nutr Soc 2010;69:103–18.	Not a research paper
Miyake Y, Sasaki S, Tanaka K, Hirota Y. Dairy food, calcium and vitamin D intake in pregnancy, and wheeze and eczema in infants. Eur Respir J 2010;35:1228–34.	Not relevant to the research question – wrong outcome
Myhre R, Brantsaeter AL, Myking S et al. Intake of probiotic food and risk of spontaneous preterm delivery. Am J Clin Nutr 2011;93:151–7.	Not relevant to the research question – outcome
Oken E, Ning Y, Rifas-Shiman SL, Rich-Edwards JW, Olsen SF, Gillman MW. Diet during pregnancy and risk of preeclampsia or gestational hypertension. Ann Epidemiol 2007;17:663–8.	Not relevant to the research question – outcome
Olafsdottir AS, Skuladottir GV, Thorsdottir I, Hauksson A, Steingrimsdottir L. Maternal diet in early and late pregnancy in relation to weight gain. Int J Obes (Lond) 2006;30:492–9.	Not relevant to the research question – outcome
Salmenhaara M, Uusitalo L, Uusitalo U et al. Diet and weight gain characteristics of pregnant women with gestational diabetes. Eur J Clin Nutr 2010;64:1433–40.	Not healthy
Stuebe AM, Oken E, Gillman MW. Associations of diet and physical activity during pregnancy with risk for excessive gestational weight gain. Am J Obstet Gynecol 2009;201:58.	Not relevant to the research question – outcome
Willers SM, Devereux G, Craig LC et al. Maternal food consumption during pregnancy and asthma, respiratory and atopic symptoms in 5-year-old children. Thorax 2007;62:773–9.	Not relevant to the research question – outcome
Yin J, Dwyer T, Riley M, Cochrane J, Jones G. The association between maternal diet during pregnancy and bone mass of the children at age 16. Eur J Clin Nutr 2010;64:131–7.	Not relevant to the research question – outcome
